# A high salt meal does not impair cerebrovascular reactivity in healthy young adults

**DOI:** 10.14814/phy2.14585

**Published:** 2020-10-10

**Authors:** Kamila U. Migdal, Austin T. Robinson, Joseph C. Watso, Matthew C. Babcock, Shannon L. Lennon, Christopher R. Martens, Jorge M. Serrador, William B. Farquhar

**Affiliations:** ^1^ Department of Kinesiology & Applied Physiology University of Delaware Newark DE USA; ^2^ Department of Pharmacology, Physiology & Neuroscience Rutgers New Jersey Medical School Newark NJ USA

**Keywords:** cerebrovascular reactivity, dietary sodium, flow mediated dilation, reactive oxygen species, transcranial Doppler ultrasound

## Abstract

A high sodium (Na^+^) meal impairs peripheral vascular function. In rodents, chronic high dietary Na^+^ impairs cerebral vascular function, and in humans, habitual high dietary Na^+^ is associated with increased stroke risk. However, the effects of acute high dietary Na^+^ on the cerebral vasculature in humans are unknown. The purpose of this study was to determine if acute high dietary Na^+^ impairs cerebrovascular reactivity in healthy adults. Thirty‐seven participants (20F/17M; 25 ± 5 years; blood pressure [BP]: 107 ± 9/61 ± 6 mm Hg) participated in this randomized, cross‐over study. Participants were given a low Na^+^ meal (LSM; 138 mg Na^+^) and a high Na^+^ meal (HSM; 1,495 mg Na^+^) separated by ≥ one week. Serum Na^+^, beat‐to‐beat BP, middle cerebral artery velocity (transcranial Doppler), and end‐tidal carbon dioxide (P_ET_CO_2_) were measured pre‐ (baseline) and 60 min post‐prandial. Cerebrovascular reactivity was assessed by determining the percent change in middle cerebral artery velocity to hypercapnia (via 8% CO_2_, 21% oxygen, balance nitrogen) and hypocapnia (via mild hyperventilation). Peripheral vascular function was measured using brachial artery flow‐mediated dilation (FMD). Changes in serum Na^+^ were greater following the HSM (HSM: Δ1.6 ± 1.2 mmol/L vs. LSM: Δ0.7 ± 1.2 mmol/L, *p* < .01). Cerebrovascular reactivity to hypercapnia (meal effect: *p* = .41) and to hypocapnia (meal effect: *p* = .65) were not affected by the HSM. Contrary with previous findings, FMD was not reduced following the HSM (meal effect: *p* = .74). These data suggest that a single high Na^+^ meal does not acutely impair cerebrovascular reactivity, and suggests that despite prior findings, a single high Na^+^ meal does not impair peripheral vascular function in healthy adults.

## Graphical abstract

A single high sodium meal compared to a low sodium meal does not impair cerebrovascular reactivity to hypercapnia or hypocapnia in healthy adults. MCA, Middle Cerebral Artery.

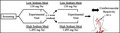

**Abbreviations**
BP: blood pressure
CO_2_: carbon dioxide
HSM: high sodium meal
LSM: low sodium meal
MAP: mean arterial pressure
MCA: middle cerebral artery
Na^+^: sodium
P_ET_CO_2_: end‐tidal carbon dioxide
TCD: transcranial Doppler

## 1 INTRODUCTION

The American Heart Association recommends 1,500 mg of dietary sodium (Na^+^) per day for optimal health (Whelton et al., 2012). Roughly nine‐in‐ten Americans consume more dietary Na^+^ than is recommended (U.S. Department of Health and Human Services and U.S. Department of Agriculture, 2015). One third of Americans report eating out at a restaurant at least once a day (Byrd et al., 2018), where individual meals may exceed the daily dietary Na^+^ intake recommendation (Auchincloss et al., 2014). Furthermore, average Na^+^ density in meals prepared at home exceeds the optimal Na^+^ recommendation (Lin, 2014). This is problematic as high dietary Na^+^ consumption increases the risk of strokes (Gardener et al., 2012), and higher serum Na^+^ concentrations are associated with ischemic stroke (Farahmand et al., 2013). Despite this link between high dietary Na^+^ and stroke risk, previous research has not examined the effects of acute high dietary Na^+^ on cerebrovascular function.

The use of transcranial Doppler (TCD) ultrasound to measure middle cerebral artery (MCA) velocity is a widely used and safe clinical research technique (Purkayastha & Sorond, 2012) and can be used during experimental conditions to assess cerebrovascular reactivity to hypercapnia (high concentrations of carbon dioxide CO_2_) and hypocapnia (low concentrations of CO_2_; McDonnell et al., 2013). Assessing cerebrovascular reactivity to hypercapnia and hypocapnia is commonly used in research and clinical settings to evaluate the vasomotor range (Ringelstein et al., 1988). Cerebrovascular reactivity to hypercapnia is a clinically important measure as cross‐sectional data has revealed that patients with diabetes (Petrica et al., 2007), hypertension (Settakis et al., 2003), and cognitive decline (Glodzik et al., 2013) demonstrate impaired cerebrovascular reactivity to hypercapnia. Longitudinal data also indicate that impaired cerebrovascular reactivity to hypercapnia is a predictor of ischemic stroke (Markus & Cullinane, 2001; Silvestrini et al., 2000). While the effects of high dietary Na^+^ intake on cerebrovascular reactivity to a vasodilator stimuli in healthy young adults are unclear, rodents fed a chronic high Na^+^ diet exhibit diminished flow‐induced dilation (Matic et al., 2018) and acetylcholine‐induced dilation of the middle cerebral artery (MCA; Durand & Lombard, 2013), independent of changes in resting arterial blood pressure (BP). Acute high dietary Na^+^ (i.e. a single meal) elevates serum Na^+^ (Migdal et al., 2020) and reduces peripheral vascular endothelial function, independent of concomitant changes in BP in humans (Blanch et al., 2015; Dickinson et al., 2011). Given the high dietary Na^+^ consumption in single sittings (Lin, 2014) and the relation between acute high Na^+^ and peripheral vascular function (Blanch et al., 2015; Dickinson et al., 2011), we decided to examine the impact of acute high Na^+^ on cerebrovascular reactivity.

Therefore, the purpose of this study was to assess cerebrovascular and peripheral vascular function following a high Na^+^ meal (HSM) compared to a low Na^+^ meal (LSM) in healthy young adults. We hypothesized that the HSM would reduce cerebrovascular reactivity compared to the LSM. As an exploratory analysis, we measured the impact of acute dietary Na^+^ on reactive oxygen species. We have previously demonstrated that chronic high dietary salt increases oxidative stress in the periphery (Greaney et al., 2012; Ramick et al., 2019) and increases in oxidative stress are associated with cerebrovascular disease (Chrissobolis & Faraci, 2008).Determining the effect of dietary Na^+^ on cerebrovascular reactivity has important implications in elucidating the link between high dietary Na^+^ consumption and increased cerebrovascular disease and stroke risk (Farahmand et al., 2013; Gardener et al., 2012).

## 2 METHODS

All procedures and protocols employed conformed to the standards set by the latest revisions of the Declaration of Helsinki of 1975, as revised in 2008 and were approved by the University of Delaware Institutional Review Board (1178955‐5). The data reported here were part of a registered clinical trial (ClinicalTrials.gov Identifier: NCT03564262). We obtained written and verbal informed consent from all participants prior to participation.

### 2.1 Study participants

Thirty‐seven participants were enrolled in the study and completed the cerebrovascular reactivity measurements. Twenty‐one participants completed the brachial artery flow‐mediated dilation (FMD) assessment. We measured reactive oxygen species in 17 participants.

During the screening visit, all participants underwent a medical history screening. Height (cm) and weight (kg) were measured for calculation of body mass index (BMI; kg/m^2^). Seated BP was measured via oscillometric assessment in triplicate following ≥ five minutes of seated rest (Welch Allyn Spot LXi). The average of the triplicate measures is reported here (Table 1). Inclusion criteria for this study included: age between 18–45 years, resting systolic BP between 90–139 mm Hg, resting diastolic BP between 50–89 mm Hg, and BMI below 30 kg/m^2^. We excluded participants who smoked or used nicotine products, anyone with a history of heart disease, cancer, metabolic disease, kidney disease, and/or other chronic diseases. We also excluded female participants who were pregnant or planning to become pregnant.

**TABLE 1:** Screening characteristics

	Average ± *SD*	Range
Number (F/M)	37 (20/17)	
Age (yr)	25 ± 5	18–33
Body mass (kg)	70 ± 12	50–81
Body mass index (kg/m^2^)	23 ± 4	19–28
Systolic BP (mm Hg)	107 ± 9	94–123
Diastolic BP (mm Hg)	61 ± 6	51–77

Note

Data presented as mean ± *SD*.

Abbreviation: BP, blood pressure.

### 2.2 Acute sodium intervention

In a randomized, double‐blind, cross‐over design, 37 participants completed the study during two experimental visits to the laboratory. Visits were separated by at least one week for male participants. Female participants were tested during the early follicular phase (days 1–4) of their menstrual cycle or placebo phase of oral contraceptives to control for menstrual status (self‐report). Participants were tested at the same time of day for each experimental visit. Participants consumed meals that contained either 6 mmol (138 mg) Na^+^ (LSM: low Na^+^ meal) or 65 mmol (1,495 mg) Na^+^ (HSM; high Na^+^ meal) on two separate occasions. These Na^+^ loads were chosen to replicate previous acute Na^+^ feeding studies ( Blanch et al., 2015; Dickinson et al., 2011). An individual not involved in data acquisition and analysis added salt (Morton Table Salt; NaCl) to 1/2 cup of tomato soup (Health Valley Organic No Salt Added Tomato Soup). All other macronutrients and micronutrients were identical. Water intake during visit two was matched to intake during visit one. Participants were asked to consume a recommended (2,300 mg) Na^+^ diet (U.S. Department of Health and Human Services and U.S. Department of Agriculture, 2015) three days before their study visit and to record their food and fluid intake during this time. We asked participants to match their diet from visit one for visit two to minimize the influence of pre‐visit diet on outcome measures. Food logs were analyzed by a dietitian using Nutrient Data System for Research (University of Minnesota [NDSR, 2012]) for Na^+^ intake. Participants were asked to abstain from caffeine, exercise, and alcohol ≥24 hr and fast ≥4 hr prior to the experimental visits.

### 2.3 Experimental instrumentation

**FIGURE 1:**
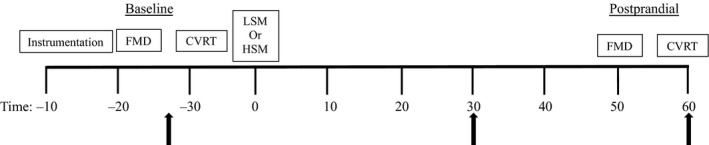
Schematic representation of the experimental visit. Following instrumentation of the participants, we performed the FMD and cerebrovascular reactivity test for the baseline assessment. Next we provided the participants with a low sodium meal (138 mg Na^+^) or a high sodium meal (1,495 mg Na^+^). We waited 50 min and then repeated the FMD test and afterwards the cerebrovascular reactivity test. Arrows indicate blood draws. Blood draws were taken at baseline, 30 min postprandial (for the assessment of reactive oxygen species) and 60 min postprandial. CVRT, cerebrovascular reactivity test; FMD, flow‐mediated dilation; HSM, high sodium meal; LSM, low sodium meal

Figure 1 presents the timing of the experimental visit. Upon arrival to the laboratory, body mass (Tanita Body Composition Analyzer, Model TBF‐300A; Arlington Heights, IL) was measured. Female participants provided a spot urine sample to confirm that they were not pregnant (hcG cassettes, Moore Medical). An intravenous catheter was placed for blood sampling throughout the visit. While supine, beat‐to‐beat BP was monitored at the middle finger of the participant's hand using photoplethysmography (Finometer; Finapres Medical Systems), heart rate was monitored through single lead ECG (lead II; Dash 2000, GE Medical Systems), and end tidal CO_2_ (P_ET_CO_2_) was sampled from a nasal cannula and measured by a gas analyzer (Capnocheck Plus Capnograph, Smiths Medical). Subjects were asked to breathe in and out through their nose. A 2‐MHz TCD ultrasound (Multidop T, DWL) probe was used to assess right MCA blood flow velocity (MCAv). The basal portion of the MCA was identified by insonating over the temporal bone above the front of the ear. Optimal signals were obtained by adjusting the insonation angle and the depth of the signal (40–60 mm), and the probe was secured with a headband throughout the protocol to secure the position and angle. The location of the TCD probe was recorded to ensure exact placement in subsequent visits. A single investigator was responsible for the placement of the TCD probe for all study participants.

### 2.4 Cerebrovascular reactivity

**FIGURE 2:**
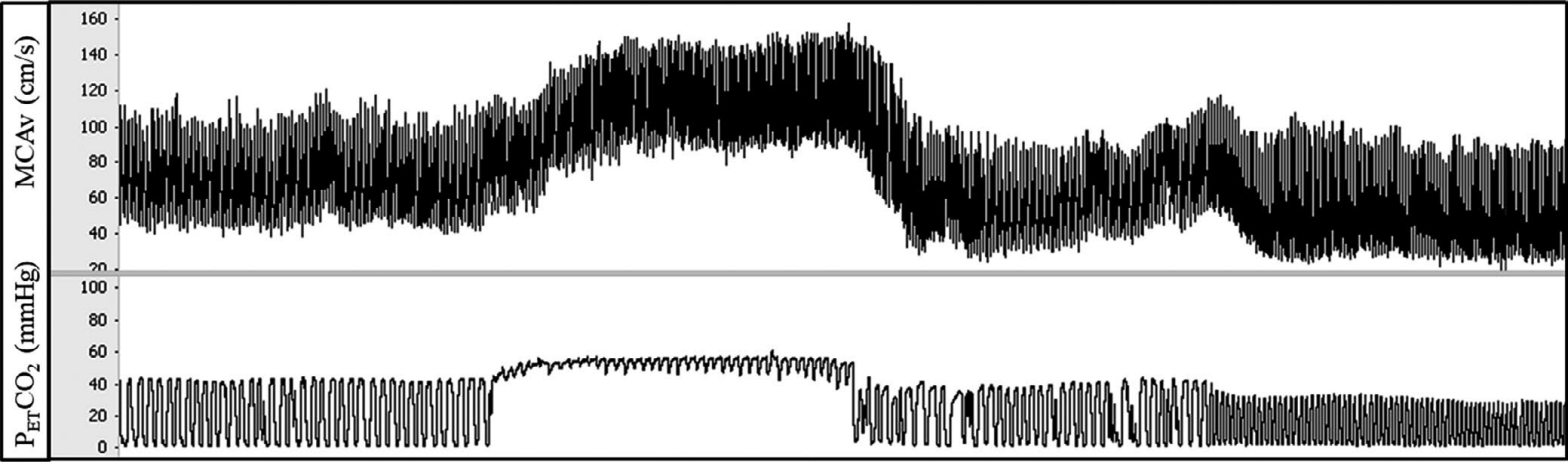
Original recording: an original recording of middle cerebral artery velocity (MCAv; top) and end‐tidal carbon dioxide levels (P_ET_CO_2_; bottom) during a cerebrovascular reactivity test. Following baseline, participants inhaled 8% CO_2_, 21% O_2_, balance N for two minutes, causing an increase in MCAv. After two minutes of recovery, participants hyperventilated for two minutes, causing a decrease in MCAv

For the cerebrovascular reactivity test, participants breathed room air at their own pace for two minutes while supine. Next, a facemask was placed over their mouth and nose to deliver 8% CO_2_, 21% O_2_, balance N_2_ (i.e., hypercapnia) for two minutes. This was followed by two minutes of recovery and ended with two minutes of mild hyperventilation to lower P_ET_CO_2_ to ~25 mm Hg (i.e., hypocapnia), as previously described (Deegan et al., 2009; Falvo et al., 2018; Serrador & Freeman, 2017; Serrador et al., 2005). Participants hyperventilated at their own pace, but study staff monitored their pace to ensure that P_ET_CO_2_ was lowered to ~25 mm Hg. We present an original recording in Figure 2. To ensure steady state conditions for the cerebrovascular reactivity test, variables for analyses were averaged over the last minute of baseline (room air), hypercapnia, recovery (room air), and hypocapnia. Cerebrovascular reactivity was calculated as the percent change from baseline in MCAv relative to the absolute change from baseline in P_ET_CO_2_. Cerebrovascular reactivity to hypercapnia and hypocapnia were analyzed separately. In the analysis, the recovery period was used as the baseline for hypocapnia. Hypercapnia leads to vasodilation of downstream resistance arterioles and increases MCAv, while hypocapnia leads to vasoconstriction of downstream resistance arterioles and decreases MCAv (Kety & Schmidt, 1946; Wasserman & Patterson, 1961). Cerebrovascular conductance index was calculated as MCAv/MAP. We calculated cerebrovascular conductance to account for changes in MAP during hypercapnia and hypocapnia. The cerebrovascular reactivity test was completed at baseline and 60‐min following the acute meal consumption for all 37 participants.

### 2.5 Peripheral vascular function

In addition to cerebrovascular assessments, 26 participants completed a peripheral vascular function assessment using brachial artery FMD (Corretti et al., 2002; Thijssen et al., 2019). Due to technical difficulties (i.e. inadequate image quality or movement during the protocol), we present 21 complete data sets. A previous study using similar dietary Na^+^ concentrations observed a reduction in FMD up to 60‐min postprandial (Blanch et al., 2015; Dickinson et al., 2011). We added this measurement to attempt to replicate those results and to determine if acute high dietary Na^+^ causes similar responses in the peripheral and cerebral vasculature. While participants were supine, the right arm was extended perpendicular to the body at heart level. An occlusion cuff connected to a rapid cuff inflator (AG101 Rapid Cuff Inflator; Hokanson) was placed on the forearm below the antecubital crease. A 12‐MHz linear phased‐array ultrasound transducer (GE P5; Healthcare) was used to acquire longitudinal images of the brachial artery and continuous Doppler blood velocity. Following one minute of baseline imaging, the cuff was inflated to 200 mm Hg for five minutes. The data were recorded continuously through the inflation period and for three minutes following cuff deflation. The cuff deflation causes a rapid increase in blood flow, increasing laminar shear stress on the vessel wall inducing vasodilation (Corretti et al., 2002). The FMD response was calculated as the percent change in the baseline diameter of the artery to the peak diameter following cuff deflation. The FMD was analyzed off‐line using commercial wall‐tracking software (Cardiovascular Suite). Each video file was analyzed by a blinded investigator. As a quality control measure, we further analyzed the FMD using a custom excel spreadsheet. The excel sheet allows the investigator to assess the diameter and velocity second‐by‐second and to verify all analysis parameters. The FMD was performed prior to soup consumption and 50 min postprandial (prior to the postprandial cerebrovascular reactivity test).

### 2.6 Blood analysis

Venous blood samples were collected prior to the meal (baseline) and 60 min postprandial and were analyzed for hemoglobin (Hb 201+ model; HemoCue), hematocrit (Pre‐calibrated Clay Adams, Readacrit Centrifuge, Becton Dickinson), serum electrolyte concentrations (Easy Electrolyte Analyzer; Medica), and plasma osmolality (3D3 Osmometer, Advanced Instruments). Participants laid in the supine position for at least 20 min following the insertion of the intravenous catheter before baseline venous blood samples were collected.

### 2.7 Reactive oxygen species

We measured parameters of oxidative stress from the venous blood sample from 17 subjects collected at three time points: prior to the meal, 30 min post and 60 min postprandial. A subset of participants (*n* = 8) completed a time control visit (no meal) where we measured reactive oxygen species at the same three time points. Reactive oxygen species were measured in whole blood using electron paramagnetic resonance (EPR) spectroscopy. After blood collection, the whole blood sample was incubated for one hour at 37°C with a superoxide‐sensitive EPR spin probe, 1‐hydroxy‐3‐methoxycarbonyl‐2,2,5,5‐tetramethylpyrrolidine (CMH). CMH was added to each whole blood sample immediately after the collection (prior to the meal, 30 min post and 60 min postprandial) and incubated. Although CMH has the highest interaction with superoxide, it is not 100% specific for superoxide as there is evidence that it reacts with peroxyl radical, peroxynitrite, and nitrogen dioxide (Berg et al., 2014; Dikalov et al., 2007). Thus, we report concentrations of reactive oxygen species. The spin probe results in the formation of stable nitroxide radicals that can be detected by EPR spectroscopy, and the amount of nitroxide formed is proportional to the concentration of reactive oxygen species. We flash froze (using liquid nitrogen) whole blood samples of 100 µl in a 1 ml syringe between buffer solutions to form a continuous frozen plug. We used Krebs HEPES buffer (noxygen Science Transfer & Diagnostics GmbH). Samples were stored at −80°C. All EPR measurements were performed at the University of Nebraska Medical Center's EPR Spectroscopy Core with a Bruker eScan EPR spectrometer (Bruker Corporation) and expressed as EPR arbitrary units.

### 2.8 Statistical analysis

The primary outcome for the statistical analysis was the effect of HSM compared with LSM on cerebrovascular reactivity to hypercapnia. We performed an a priori power analysis based on pilot data collection to determine the number of participants needed to detect an effect size of 0.67. We determined that a total of 20 subjects would be needed to provide 90% power with alpha set at 0.05 to detect a significant main effect of the meal on cerebrovascular reactivity to hypercapnia. Data were acquired at 1,000 Hz (LabChart 8.0 Pro, ADInstruments) and analyzed off‐line with a custom MATLAB script (Mathworks). Data were analyzed using two‐way repeated measures ANOVA (meal × time). Due to missing samples, we analyzed the blood results using a generalized linear mixed‐model analysis with repeated measures for meal and time. We explored potential sex differences using a three‐way ANOVA (sex × meal × time; Table 5). Tukey's post hoc comparisons were made when appropriate. The hemodynamic variables during the cerebrovascular reactivity test were analyzed using a repeated measure one‐way ANOVA (Table 3). Average Na^+^ intake and energy intake from the dietary food logs was analyzed using a paired *t* test. Reactive oxygen species during the time control visit was analyzed using a repeated measure one‐way ANOVA. Data are expressed as mean ± *SD* with alpha set at 0.05. Statistical analyses were completed using GraphPad Prism Version 8.0.

## 3 RESULTS

Participant screening characteristics are presented in Table 1. All participants were non‐hypertensive and non‐obese. All participants fell within the American Heart Association criteria for non‐hypertensive blood pressure (Whelton et al., 2018; Table 1). Average dietary Na^+^ intake for three days prior to each experimental visit, as quantified from the self‐report dietary food logs, was comparable across visits (Visit 1:2,260 ± 842 mg Na^+^, Visit 2:2,357 ± 900 mg Na^+^, *p* = .57). Average caloric intake was also comparable across visits (Visit 1:1,770 ± 561 kcal, Visit 2:1,708 ± 440 kcal, *p* = .99).

### 3.1 Response to the meal condition

**FIGURE 3:**
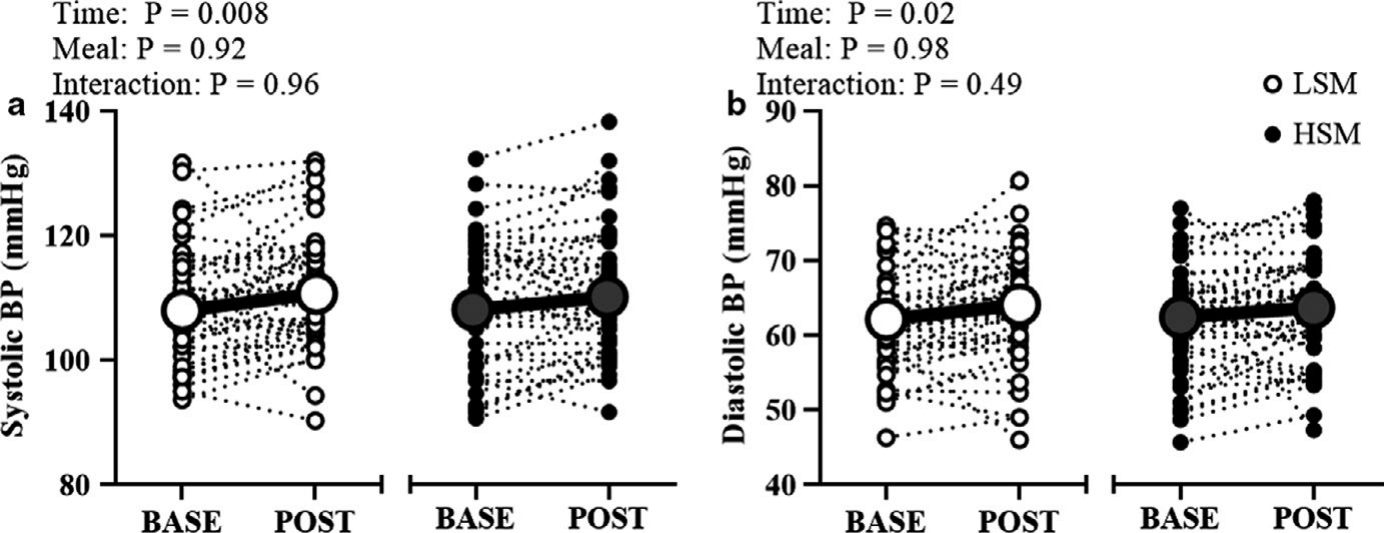
Blood pressure at baseline and 60 min postprandial. Two‐way RM ANOVA; Systolic (a) and diastolic (b) blood pressure modestly increased following both experimental visits but there was no main effect of the meal. BASE, baseline; BP, blood pressure; HSM, high sodium meal; LSM, low sodium meal; POST, postprandial

The biochemical variables are presented in Table 2. Both the LSM and HSM increased postprandial serum Na^+^ concentrations (time: *p* < .001, meal: *p* = .03, interaction: *p* = .02) and plasma osmolality (time: *p* < .001, meal: *p* = .88, interaction: *p* = .004) compared with baseline. The significant interaction indicates a greater overall increase observed following the HSM. In this regard, postprandial serum Na^+^ concentrations were higher 60‐min (HSM: Δ1.6 ± 1.2 mmol/L vs. LSM: Δ0.7 ± 1.2 mmol/L) following ingestion of the HSM compared to the LSM. There was no main effect of the meal for hematocrit (*p* = .90) and hemoglobin (*p* = .45) suggesting that the HSM did not cause any acute expansion of plasma volume. Postprandial systolic (time: *p* = .008) and diastolic (time: *p* = .02) BP increased slightly following each meal; however, there was no main effect of the meal on BP and no interaction between the meal and the postprandial increase in BP (Figure 3). Although there was a main effect of sex on systolic BP (*p* < .001) where male individuals had higher systolic BP then female individuals, there was no interaction (sex × meal × time interaction: *p* = .53) or main effect of the meal (*p* = .27). There were no sex differences in diastolic BP (Table 5).

**TABLE 2:** Biochemical variables following the acute sodium intervention

	LSM		HSM		*p*‐value			
	BASE	POST	BASE	POST	Time	Meal	Time × meal	
Serum Na^+^ (mmol/L)	139.6 ± 2.0	140.4 ± 1.8*	139.8 ± 1.9	141.5 ± 2.2^*^	.001	.03		.02
Serum K^+^ (mmol/L)	3.9 ± 0.3	4.0 ± 0.3	3.9 ± 0.3	4.1 ± 0.4	.008	.83		.15
Serum Cl^−^ (mmol/L)	104.2 ± 2.1	104.5 ± 2.2*	104.7 ± 1.9	105.8 ± 1.9^*^	<.001	.001		.002
Plasma osmolality (mOsm/kg H_2_O)	290.1 ± 4.1	291.6 ± 4.0*	289.6 ± 3.7	292.8 ± 4.0*	<.001	.88		.004
Hematocrit (%)	40.8 ± 3.7	41.4 ± 3.2	41.1 ± 3.3	41.2 ± 3.3	.05	.90		.10
Hemoglobin (g/dl)	13.0 ± 1.2	13.3 ± 1.3	13.2 ± 1.4	13.3 ± 1.5	.70	.45		.45

Note

Generalized linear mixed‐model analysis. Data presented as mean ± *SD*.

Abbreviations: BASE, baseline; Cl^−^, chloride; HSM, high Na^+^ meal; K^+^, potassium; LSM, low Na^+^ meal; Na^+^, sodium; POST: postprandial.

* *p* < .05 versus respective baseline.

^†^ *p* < .05 versus LSM; *N* = 37.

### 3.2 Cerebrovascular reactivity

**FIGURE 4:**
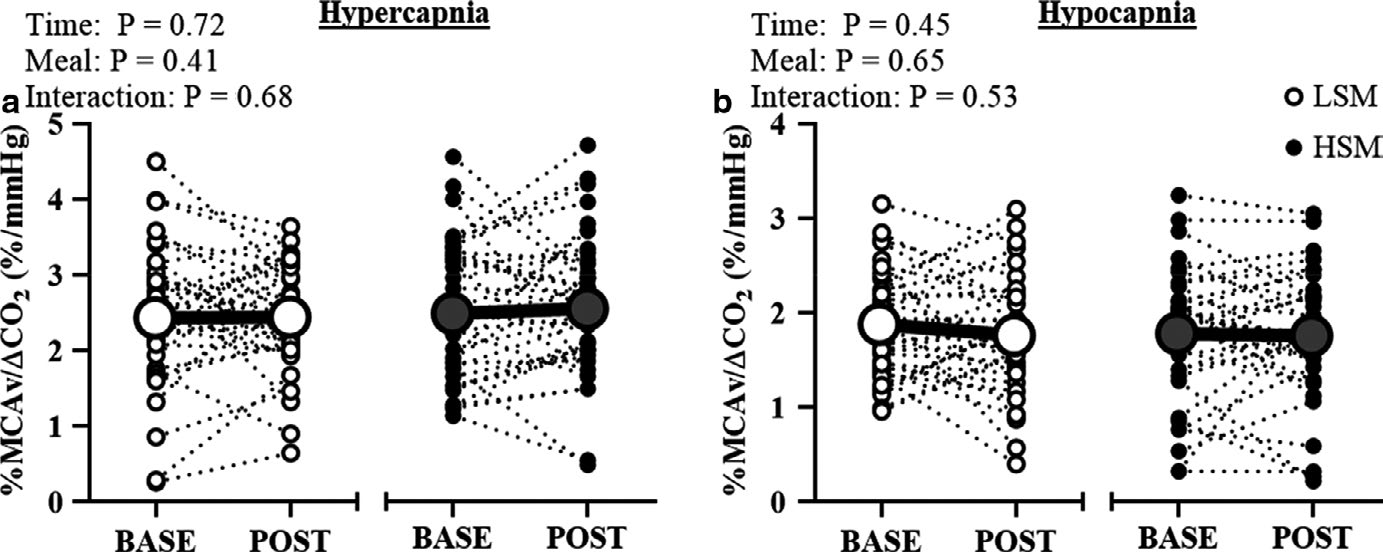
Cerebrovascular reactivity following the acute sodium intervention. Two‐way RM ANOVA. Cerebrovascular reactivity to hypercapnia (a) and to hypocapnia (b) were not different following both meals. BASE, baseline; CO_2_, carbon dioxide; HSM, high sodium meal; LSM, low sodium meal; MCAv, middle cerebral artery velocity; POST, postprandial

**FIGURE 5:**
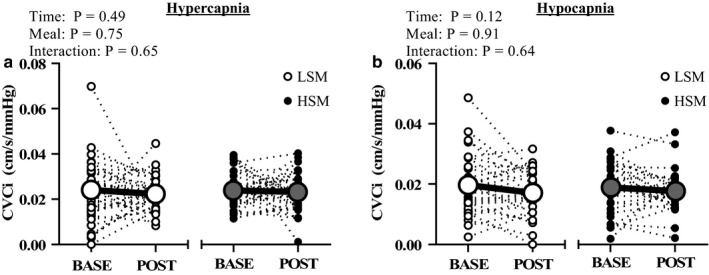
Cerebrovascular conductance following the acute sodium intervention. Two‐way RM ANOVA. Cerebrovascular conductance to hypercapnia (a) and to hypocapnia (b) were not different following both meals. BASE, baseline; CVCi, cerebrovascular conductance index; HSM, high sodium meal; LSM, low sodium meal; MCAv, middle cerebral artery velocity; POST, postprandial

Table 3 includes hemodynamic variables at rest and during the cerebrovascular reactivity test. MCAv at baseline and during the cerebrovascular reactivity test were not different following the HSM compared to LSM. P_ET_CO_2_ was not different at rest and increased during hypercapnia and decreased during hypocapnia to a similar extent in both conditions. Heart rate during hypercapnia and hypocapnia was not different between conditions. Although MAP did modestly increase from baseline to hypercapnia after both conditions, there was no main effect of the meal at any time point throughout the cerebrovascular reactivity test. When expressed as percent change, cerebrovascular reactivity to hypercapnia (Figure 4a) and to hypocapnia (Figure 4b) were not different between the two meal conditions. Cerebrovascular conductance in response to hypercapnia (Figure 5a) and to hypocapnia (Figure 5b) were also not different following the meals. There were no sex differences in the responses to hypercapnia (main effect of sex: *p* = .58; main effect of meal: *p* = .42; sex × meal × time interaction: *p* = .14) or to hypocapnia (main effect of sex: *p* = .63; main effect of meal: *p* = .66; sex × meal × time interaction: *p* = .60, Table 5). There were also no sex differences in cerebrovascular conductance during hypercapnia or hypocapnia (Table 5).

**TABLE 3:** Hemodynamic variables during the cerebrovascular reactivity test

	LSM		HSM		*p*‐value		
	BASE	POST	BASE	POST	Time	Meal	Time × meal
Rest							
MCAv_mean_ (cm/s/mm Hg)	64 ± 11	66 ± 11	64 ± 9	67 ± 11	.004	.40	.26
P_ET_CO_2_ (mm Hg)	42 ± 4	43 ± 2	43 ± 3	43 ± 3	.33	.32	.32
Heart Rate (bpm)	59 ± 12	59 ± 12	56 ± 11	57 ± 12	.03	.32	.21
MAP (mm Hg)	88 ± 7	90 ± 7	86 ± 7	90 ± 7	.01	.26	.24
Hypercapnia							
MCAv_mean_ (cm/s/mm Hg)	96 ± 22*	98 ± 19*	94 ± 17*	100 ± 18*	.007	.28	.28
P_ET_CO_2_ (mm Hg)	60 ± 6*	60 ± 4*	60 ± 4*	60 ± 4*	.17	.33	.33
Heart Rate (bpm)	66 ± 14*	68 ± 14*	65 ± 14*	67 ± 12*	.002	.48	.21
MAP (mm Hg)	93 ± 6*	94 ± 7*	90 ± 9*	94 ± 7*	.004	.52	.20
Recovery							
MCAv_mean_ (cm/s/mm Hg)	57 ± 11*	58 ± 12*	58 ± 10*	57 ± 9*	.45	.96	.83
P_ET_CO_2_ (mm Hg)	40 ± 5*	40 ± 5*	40 ± 4*	41 ± 4*	.35	.34	.34
Heart Rate (bpm)	60 ± 13*	61 ± 12*	59 ± 11*	60 ± 12*	.80	.65	.37
MAP (mm Hg)	88 ± 11	92 ± 9	89 ± 10	91 ± 13	.005	.38	.76
Hypocapnia							
MCAv_mean_ (cm/s/mm Hg)	41 ± 9^*^	42 ± 10^*^	40 ± 7^*^	42 ± 9^*^	.02	.44	.32
P_ET_CO_2_ (mm Hg)	29 ± 3^*^	30 ± 4^*^	28 ± 3^*^	29 ± 3^*^	.35	.33	.34
Heart Rate (bpm)	70 ± 16^*^	71 ± 16^*^	68 ± 13^*^	71 ± 14^*^	.01	.72	.97
MAP (mm Hg)	86 ± 7^*^	89 ± 7^*^	85 ± 8^*^	87 ± 7^*^	.003	.28	.76

Note

Data presented as mean ± *SD*. Each variable was analyzed using a Two‐way RM ANOVA (meal × time). The *p*‐values indicate the results from the Two‐Way RM ANOVA (meal × time). The CVR test was separately analyzed using a RM One‐Way ANOVA for each time point of the test (rest, hypercapnia, recovery, hypocapnia).

Abbreviations: BASE, baseline; HSM, high Na^+^ meal; LSM, low Na^+^ meal; MAP, mean arterial pressure; MCA_Vmean_, middle cerebral artery mean velocity; P_ET_CO_2_, end‐tidal carbon dioxide; POST, postprandial.

* *p* < .05 versus rest.

^†^ *p* < .05 versus recovery.

### 3.3 Peripheral vascular function

**FIGURE 6:**
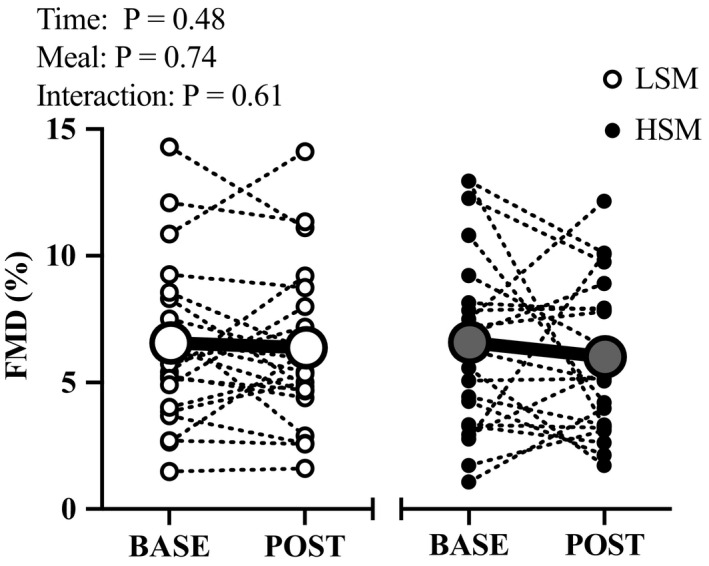
Brachial artery flow‐mediated dilation following the acute sodium intervention was not different following both meals. BASE, baseline; FMD, flow‐mediated dilation; HSM, high sodium meal; LSM, low sodium meal; POST, postprandial; *N* = 21. Two‐way RM ANOVA

We measured brachial artery FMD in 21 participants. Brachial artery diameters measured during the one‐minute baseline period were similar between visits at baseline and postprandial (time: *p* = .48, meal: *p* = .81, interaction: *p* = .30; Table 4). The FMD response was not different following the acute Na^+^ intervention (HSM: Base = 6.06 ± 3.5, Post = 5.88 ± 3.1 vs. LSM: Base = 6.16 ± 3.1, Post = 5.88 ± 3.27%; Figure 6). There were no differences when normalizing the FMD to shear rate (HSM: Base = 0.5 ± 0.9, Post = 0.5 ± 0.9 vs. LSM: Base = 0.5 ± 0.8, Post = 0.5 ± 0.6 FMD%/AUC 10^3^, main effect of meal: *p* = .38). There were no sex differences in the FMD response (main effect of sex: *p* = .92; main effect of meal: *p* = .63; sex x meal x time interaction: *p* = .28, Table 5).

**TABLE 4:** Brachial artery flow‐mediated dilation parameters

Test parameters	LSM		HSM		*p*‐value		
	BASE	POST	BASE	POST	Time	Meal	Time × meal
Baseline brachial artery diameter (mm)	3.5 ± 0.4	3.5 ± 0.7	3.5 ± 0.7	3.5 ± 0.6	.48	.81	.30
Peak diameter (mm)	3.7 ± 0.5	3.8 ± 0.7	3.8 ± 0.7	3.7 ± 0.6	.85	.20	.83
Diameter change (mm)	0.20 ± 0.13	0.18 ± 0.10	0.23 ± 0.10	0.20 ± 0.10	.09	.41	.59
Peak shear rate (s^−1^)	984 ± 502	929 ± 366	1,014 ± 669	1,008 ± 662	.27	.44	.21
Shear rate (AUC)	31,485 ± 15,340	29,033 ± 14,314	34,364 ± 13,138	29,588 ± 18,291	.08	.48	.58

Note

Data presented as mean ± *SD*. Two‐way RM ANOVA.

Abbreviations: AUC, area under curve; BASE, baseline; HSM, high Na^+^ meal; LSM, low Na^+^ meal; POST, postprandial.

**TABLE 5:** Sex differences

	Female				Male				*p*‐value						
	LSM		HSM		LSM		HSM								
	BASE	POST	BASE	POST	BASE	POST	BASE	POST	Time	Sex	Meal	Time × sex	Time × meal	Sex × meal	Time × sex × meal
Systolic BP (mm Hg)	105 ± 7	106 ± 8	103 ± 8	105 ± 6	115 ± 7*	116 ± 8*	118 ± 8*	119 ± 9*	.25	<.001	.27	.93	.73	.03	.53
Diastolic BP (mm Hg)	62 ± 6	63 ± 8	63 ± 8	64 ± 6	61 ± 7	64 ± 7	63 ± 8	66 ± 8	.17	.70	.07	.54	.84	.23	.88
CVRT: *Hypercapnia* (%/mm Hg)	2.4 ± 1.0	2.4 ± 0.9	2.5 ± 1.0	2.4 ± 1.1	2.5 ± 0.8	2.5 ± 0.6	2.5 ± 0.8	2.7 ± 0.7	.72	.58	.42	.75	.61	.88	.14
CVRT: *Hypocapnia* (%/mm Hg)	1.9 ± 0.5	1.8 ± 0.7	1.8 ± 0.8	1.8 ± 0.7	1.8 ± 0.5	1.7 ± 0.4	1.7 ± 0.7	1.7 ± 0.7	.43	.63	.66	.51	.51	.91	.60
CVCi: *Hypercapnia* (cm/s/mm Hg)	0.02 ± 0.02	0.02 ± 0.01	0.02 ± 0.01	0.02 ± 0.01	0.02 ± 0.01	0.02 ± 0.01	0.03 ± 0.01	0.03 ± 0.01	.54	.27	.70	.40	.68	.24	.49
CVCi: *Hypocapnia* (cm/s/mm Hg)	0.02 ± 0.01	0.02 ± 0.02	0.02 ± 0.01	0.02 ± 0.01	0.02 ± 0.01	0.01 ± 0.01	0.02 ± 0.01	0.01 ± 0.01	.10	.82	.89	.20	.62	.77	.51
FMD (%)	6.0 ± 2.3	5.8 ± 3.6	5.6 ± 4.1	5.9 ± 3.7	5.2 ± 4.3	5.2 ± 4.4	5.2 ± 4.5	5.1 ± 3.0	.13	.82	.63	.89	.78	.78	.30

Note

Data presented as mean ± *SD*. RM Three‐Way ANOVA.

Abbreviations: BASE, baseline; BP, blood pressure; CVCi, cerebrovascular conductance index; CVRT, cerebrovascular reactivity test; FMD, flow‐mediated dilation; HSM, high Na^+^ meal; LSM, low Na^+^ meal; POST, postprandial.

* *p* < .05.

### 3.4 Reactive oxygen species

**FIGURE 7:**
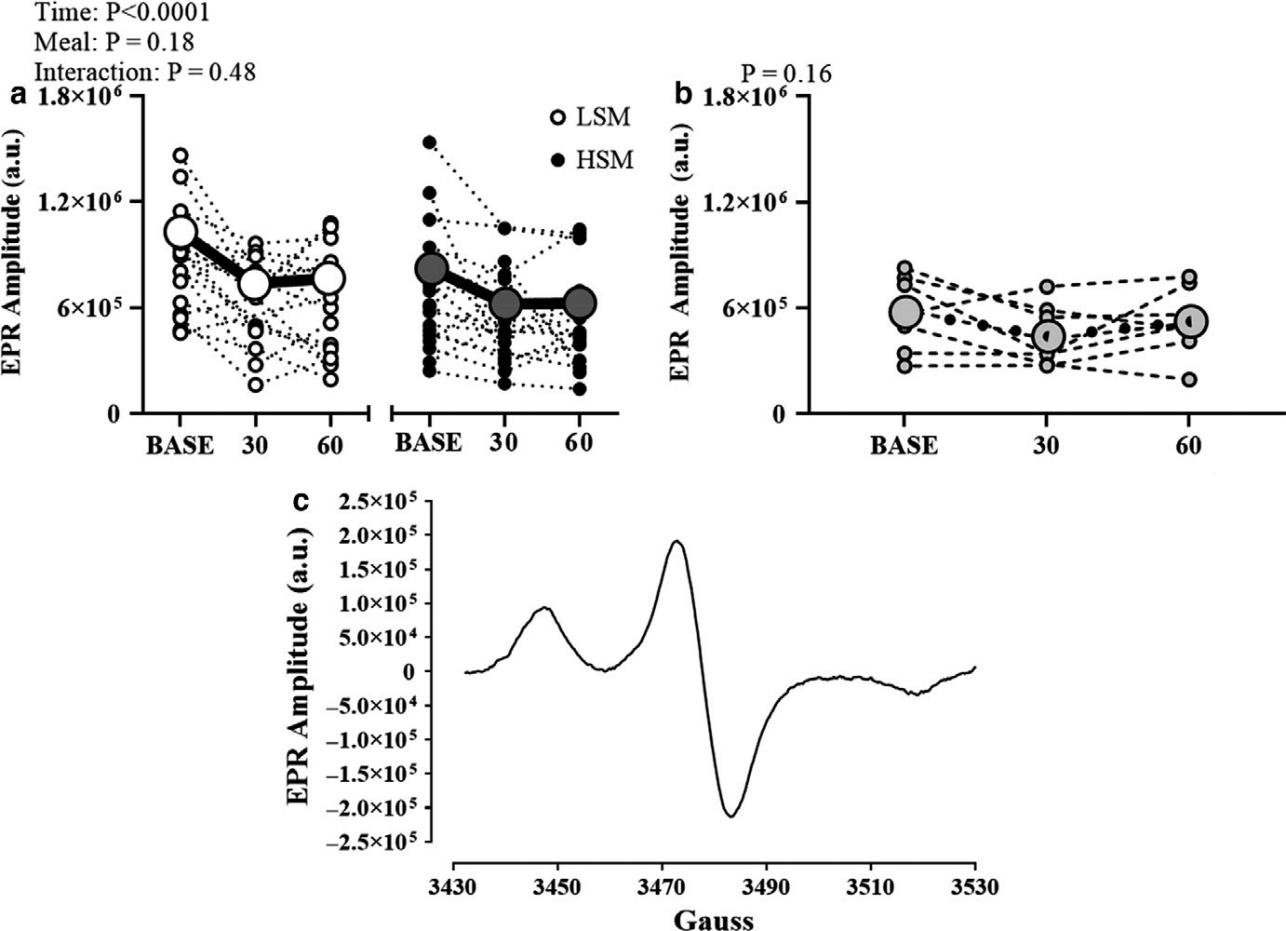
Reactive oxygen species following the acute sodium intervention (a) and the time control visit (b). There was a main effect of time for reactive oxygen species where reactive oxygen species decreased 30 and 60 min postprandial during both experimental visits. There were no differences in reactive oxygen species in the time control visit. Representative EPR spectra (c). The EPR amplitude (in arbitrary units) is directly proportional to the amount of reactive oxygen species in the sample. AU, arbitrary units; BASE, baseline; EPR, electron paramagnetic resonance; HSM, high sodium meal; LSM, low sodium meal. *N* = 17. Two‐way RM ANOVA (a); One‐way RM ANOVA (b)

We measured reactive oxygen species in 17 participants. There was a main effect of the meal in the EPR amplitude (*p* < .0001) where reactive oxygen species modestly decreased from baseline in both conditions (Figure 7). For the time control data, we have eight complete data sets. There were no differences in reactive oxygen species in the time control visit (RM one‐way ANOVA *p* = .16).

## 4 DISCUSSION

Epidemiological studies suggest a link between chronic high dietary Na^+^ consumption and stroke incidence (Appel et al., 2011). Prior evidence suggests that an acute HSM causes a decline in peripheral vascular function (Blanch et al., 2015; Dickinson et al., 2011). Given the potential link between FMD and cerebrovascular reactivity (Ainslie et al., 2007; Lavi et al., 2006), we determined the impact of acute high dietary Na^+^ on cerebrovascular reactivity in adults. The primary finding of the present study is that a single HSM modestly increases serum Na^+^ but does not impair postprandial cerebrovascular reactivity to CO_2_ or peripheral vascular function in healthy young adults. There were also no differences in cerebrovascular conductance following the experimental conditions. Thus, contrary with our hypothesis, we did not detect a difference in cerebrovascular reactivity following the single HSM compared to a LSM in healthy young adults.

We measured cerebrovascular reactivity following an acute Na^+^ meal because previous data demonstrated an adverse effect of acute Na^+^ loading on peripheral vascular function (Dickinson et al., 2011), yet there were no prior data assessing cerebrovascular function following a similar acute Na^+^ meal. We hypothesized that a HSM compared to a LSM would reduce cerebrovascular reactivity which would be directionally similar to the reductions in reactivity of isolated MCA following *chronic* high Na^+^ feeding in rodents (Cosic et al., 2016; Durand & Lombard, 2013; Lombard et al., 2003; Matic et al., 2018). Based on our findings, a single HSM does not change cerebrovascular reactivity in healthy adults.

With regard to peripheral vascular function, in contrast with previous findings by Dickinson et al. (2011) and Blanch et al. (2015), we did not observe a reduction in brachial artery FMD following an acute ingestion of a HSM. Although our study used a similar concentration of Na^+^, Dickinson et al. tested participants aged 18–70 years old with a mean age of 37 years whereas our study included only younger subjects with a mean age of 25 years. Additionally, we imaged the brachial artery continuously while Dickinson et al. imaged every 15 s following cuff release (Dickinson et al., 2011). Thus, methodological differences may explain our divergent findings.

Our laboratory has previously demonstrated that seven days of high dietary Na^+^ feeding impaired nitric oxide‐mediated dilation of peripheral microvascular function which was improved by infusion of ascorbic acid, suggesting a role for oxidative stress in contributing to the impairment (Greaney et al., 2012). Contrary with our hypothesis, there was no evidence of an increase in reactive oxygen species using our acute dietary Na^+^ paradigm. In fact, we found a modest decrease in reactive oxygen species following both meal conditions, as depicted in Figure 7. This lack of an increase in reactive oxygen species is consistent with a lack of a decline of FMD and cerebrovascular reactivity following the HSM. Not surprisingly, there are differences in the physiological effects of acute versus chronic high dietary Na^+^ consumption. Previous studies provide evidence that *chronic* dietary salt consumption induces endothelial cell oxidative stress (Ramick et al., 2019) and impairs cutaneous microvascular function due to increases in oxidative stress (Greaney et al., 2012). These studies assessed reactive oxygen species following chronic dietary Na^+^ intake at the microvascular level. Additionally, previous studies used pharmacological interventions such as the addition of apocynin, tempol (Ramick et al., 2019), and ascorbic acid (Greaney et al., 2012; Ramick et al., 2019) which ameliorated any negative impacts of chronic dietary salt suggesting a role of oxidative stress. Nevertheless, an acute Na^+^ meal does not appear to increase blood reactive oxygen species in young healthy adults.

To further explore the modest decline in reactive oxygen species in both acute dietary conditions, we collected additional time control data, to see if this was a time‐dependent alteration in reactive oxygen species. Our time control data suggest that reactive oxygen species are somewhat stable over the period of assessment, which is consistent with a previous study that measured reactive oxygen species using similar techniques at three time points following a placebo‐control, and found no main effect of time when analyzing reactive oxygen species throughout the experimental visit (Jouett et al., 2016). Therefore, we do not have an explanation for the modest, yet consistent and statistically significant decline, in reactive oxygen species during both acute meal conditions, but it is clear that the Na^+^ content of the meal had no impact on systemic reactive oxygen species in healthy controls.

It is worth emphasizing that our findings apply only to young adults and the responses could differ in older adults. For example, a recent study demonstrated that postprandial hyperlipemia following a high fat meal lowered cerebrovascular reactivity to CO_2_ in older adults (67 ± 5 years) but not in young (25 ± 6 years; Marley et al., 2017). In this study, they found an increase in reactive oxygen species but no reduction in cerebrovascular reactivity to CO_2_ in the young (Marley et al., 2017). Another study examining the impact of a high fat meal on peripheral and cerebral vascular function concluded that the high fat meal impairs FMD 2 hr postprandial but not cerebrovascular reactivity in ten healthy young men (Patik et al., 2018). Although the authors did not directly measure reactive oxygen species in this study, the reduction in FMD was attributed to increases in reactive oxygen species following the high fat meal. This suggests that the cerebrovasculature may be more resilient to the effects of an acute adverse diet.

Finally, we observed a modest increase in systolic and diastolic BP following both conditions, regardless of meal. Blanch et al. (2015) used a similar acute Na^+^ condition and found increases in postprandial diastolic BP and mean BP with no effect of the dietary Na^+^ concentrations. However, Suckling et al. (2012) demonstrated that compared to the low‐Na^+^ meal, the high Na^+^ meal increased systolic BP and the increase in BP was related to postprandial increases in plasma Na^+^ concentrations. Nonetheless, we found a modest increase in systolic and diastolic BP following both conditions and thus, it is unlikely to be related to plasma Na^+^ concentrations. Additionally, we did not observe a difference in cerebrovascular conductance following the acute dietary Na^+^ meals.

### 4.1 Limitations

While this was a cross‐over design study providing insight into the acute effects of dietary Na^+^ on the cerebral vasculature, our study was not without limitations. First, we designed this study as a double‐blind study. However, it is possible that the participants could taste the difference between the two meals. Whether this influences any measurement is unclear. Nevertheless, we ensured that all investigators involved in data acquisition or analysis were blinded to the treatment condition. Additionally, MCAv is a surrogate of cerebral blood flow as the vessel diameter cannot be directly measured using TCD. TCD is a reliable method to estimate cerebral blood flow under normal physiological conditions (Serrador et al., 2000). Previous work has found that the cross‐sectional area of the MCA increased after three minutes of hypercapnia (Coverdale et al., 2015), while our hypercapnic stimulus was kept to two minutes. Nonetheless, we cannot rule out the possibility that the MCA dilated during the cerebrovascular reactivity tests, which would underestimate cerebral blood flow during hypercapnia and overestimate during hypocapnia (Coverdale et al., 2014). In addition, 8% CO_2_ caused modest increases in MAP and thus, the increases in MCAv may have been in part due to the increases in MAP. There was no main effect of the meal on these changes in MAP during the cerebrovascular reactivity test and cerebrovascular conductance was not different between conditions. There was no rest period between the FMD and cerebrovascular reactivity test. The findings of our exploratory analysis of reactive oxygen species should be interpreted with caution given the small sample size. Additionally, this study was not designed to assess sex differences and thus, additional data are required to confirm the sex differences findings (Table 5). Nevertheless, the present study is the first to examine the effects of dietary Na^+^ intake on cerebral blood flow responses to changes in CO_2_ in healthy, young adults.

## 5 CONCLUSION

This study demonstrates that in healthy young adults, cerebrovascular reactivity to hyper‐ and hypocapnia is not affected by a single high dietary Na^+^ meal. Since we only examined an acute dietary Na^+^ intervention in young individuals, future studies are needed to examine the potential impact of acute Na^+^ loading in older adults. In addition, it is not known what impact acute and chronic high dietary Na^+^ loading has on cerebral blood flow regulation in young and older adults. Older adults are likely to be more vulnerable to the effects of high dietary Na^+^. While the goal of this study was to isolate the effects of acute high dietary Na^+^ on cerebrovascular reactivity, future studies could examine typical Western meals that include high saturated fats and fructose.

## CONFLICT OF INTEREST

The authors declare no conflict of interest.

## AUTHORS CONTRIBUTIONS

KUM. ATR, JCW, MCB, JMS, and WBF contributed to study design. All authors contributed to the acquisition, analysis, and interpretation of the data. KUM drafted the manuscript. All authors revised and approved the final version of the manuscript. All persons designated as authors qualify for authorship, and all those who qualify for authorship are listed.

## References

[phy214585-bib-0001] Ainslie, P. N. , Murrell, C. , Peebles, K. , Swart, M. , Skinner, M. A. , Williams, M. J. , & Taylor, R. D. (2007). Early morning impairment in cerebral autoregulation and cerebrovascular CO2 reactivity in healthy humans: Relation to endothelial function. Experimental Physiology, 92(4), 769–777. 17384117 10.1113/expphysiol.2006.036814

[phy214585-bib-0002] Appel, L. J. , Frohlich, E. D. , Hall, J. E. , Pearson, T. A. , Sacco, R. L. , Seals, D. R. , Sacks, F. M. , Smith, S. C. Jr , Vafiadis, D. K. , & Van Horn, L. V. (2011). The importance of population‐wide sodium reduction as a means to prevent cardiovascular disease and stroke: A call to action From the American Heart Association. Circulation, 123, 1138–1143. 10.1161/CIR.0b013e31820d0793 21233236

[phy214585-bib-0003] Auchincloss, A. H. , Leonberg, B. L. , Glanz, K. , Bellitz, S. , Ricchezza, A. , & Jervis, A. (2014). Nutritional value of meals at full‐service restaurant chains. Journal of Nutrition Education and Behavior, 46(1), 75–81. 10.1016/j.jneb.2013.10.008 24369812

[phy214585-bib-0004] Berg, K. , Ericsson, M. , Lindgren, M. , & Gustafsson, H. (2014). A high precision method for quantitative measurements of reactive oxygen species in frozen biopsies. PLoS One, 9(3), e90964 10.1371/journal.pone.0090964 24603936 PMC3947958

[phy214585-bib-0005] Blanch, N. , Clifton, P. M. , Petersen, K. S. , & Keogh, J. B. (2015). Effect of sodium and potassium supplementation on vascular and endothelial function: A randomized controlled trial. The American Journal of Clinical Nutrition, 101, 939–946. 10.3945/ajcn.114.105197 25787997

[phy214585-bib-0006] Byrd, K. , Almanza, B. , Ghiselli, R. F. , Behnke, C. , & Eicher‐Miller, H. A. (2018) Reported action to decrease sodium intake is associated with dining out frequency and use of menu nutrition information among US adults. Journal of the Academy of Nutrition and Dietetics, 118(5), 824–835. 10.1016/j.jand.2017.06.012 28780237

[phy214585-bib-0007] Chrissobolis, S. , & Faraci, F. M. (2008). The role of oxidative stress and NADPH oxidase in cerebrovascular disease. Trends in Molecular Medicine, 14(11), 495–502. 10.1016/j.molmed.2008.09.003 18929509 PMC3140460

[phy214585-bib-0008] Corretti, M. C. , Anderson, T. J. , Benjamin, E. J. , Celermajer, D. , Charbonneau, F. , Creager, M. A. , Deanfield, J. , Drexler, H. , Gerhard‐Herman, M. , Herrington, D. , Vallance, P. , Vita, J. , & Vogel, R. (2002). Guidelines for the ultrasound assessment of endothelial‐dependent flow‐mediated vasodilation of the brachial artery: A report of the International Brachial Artery Reactivity Task Force. Journal of the American College of Cardiology, 39(2), 257–265. 10.1016/S0735-1097(01)01746-6 11788217

[phy214585-bib-0009] Cosic, A. , Jukic, I. , Stupin, A. , Mihalj, M. , Mihaljevic, Z. , Novak, S. , Vukovic, R. , & Drenjancevic, I. (2016). Attenuated flow‐induced dilatation of middle cerebral arteries is related to increased vascular oxidative stress in rats on a short‐term high salt diet. Journal of Physiology, 594(17), 4917–4931. 10.1113/JP272297 27061200 PMC5009804

[phy214585-bib-0010] Coverdale, N. S. et al (2015). Heterogeneous patterns of vasoreactivity in the middle cerebral and internal carotid arteries. American Journal of Physiology. Heart and Circulatory Physiology, 308(9), H1030–H1038. 25724496 10.1152/ajpheart.00761.2014

[phy214585-bib-0011] Coverdale, N. S. , Gati, J. S. , Opalevych, O. , Perrotta, A. , & Shoemaker, J. K. (2014). Cerebral blood flow velocity underestimates cerebral blood flow during modest hypercapnia and hypocapnia. Journal of Applied Physiology, 117(10), 1090–1096. 10.1152/japplphysiol.00285.2014 25012027

[phy214585-bib-0012] Deegan, B. M. , Sorond, F. A. , Lipsitz, L. A. , ÓLaighin, G. , & Serrador, J. M. (2009). Gender related differences in cerebral autoregulation in older healthy subjects. Conference Proceedings of the IEEE Engineering in Medicine and Biology Society, 2859–2862. 10.1109/IEMBS.2009.5333604 PMC2915823 19964277

[phy214585-bib-0013] Dickinson, K. M. , Clifton, P. M. , & Keogh, J. B. (2011). Endothelial function is impaired after a high‐salt meal in healthy subjects. American Journal of Clinical Nutrition, 93(3), 500–505. 10.3945/ajcn.110.006155 21228265

[phy214585-bib-0015] Dikalov, S. , Griendling, K. K. , & Harrison, D. G. (2007). Measurement of reactive oxygen species in cardiovascular studies. Hypertension, 49(4), 717–727. 10.1161/01.HYP.0000258594.87211.6b 17296874 PMC1993891

[phy214585-bib-0016] Durand, M. J. , & Lombard, J. H. (2013). Low‐dose angiotensin II infusion restores vascular function in cerebral arteries of high salt‐fed rats by increasing copper/zinc superoxide dimutase expression. American Journal of Hypertension, 26(6), 739–747. 10.1093/ajh/hpt015 23443725 PMC3697069

[phy214585-bib-0017] Falvo, M. J. , Lindheimer, J. B. , & Serrador, J. M. (2018). Dynamic cerebral autoregulation is impaired in Veterans with Gulf War Illness: A case‐control study. PLoS One, 13(10), e0205393 10.1371/journal.pone.0205393 30321200 PMC6188758

[phy214585-bib-0018] Farahmand, F. , Anzali, B. C. , Heshmat, R. , Ghafouri, H. B. , & Hamedanchi, S. (2013). Serum sodium and potassium levels in cerebro‐vascular accident patients. The Malaysian Journal of Medical Sciences: MJMS, 20(3), 39–43. PMC3743980 23966823

[phy214585-bib-0019] Gardener, H. , Rundek, T. , Wright, C. B. , Elkind, M. S. V. , & Sacco, R. L. (2012). Dietary sodium and risk of stroke in the Northern Manhattan Study. Stroke, 43(5), 1200–1205. 10.1161/STROKEAHA.111.641043 22499576 PMC3347890

[phy214585-bib-0020] Glodzik, L. , Randall, C. , Rusinek, H. , & de Leon, M. J. (2013). Cerebrovascular reactivity to carbon dioxide in Alzheimer's disease. Journal of Alzheimer's Disease, 35(3), 427–440. 10.3233/JAD-122011 PMC3776495 23478306

[phy214585-bib-0021] Greaney, J. L. , DuPont, J. J. , Lennon‐Edwards, S. L. , Sanders, P. W. , Edwards, D. G. , & Farquhar, W. B. (2012). Dietary sodium loading impairs microvascular function independent of blood pressure in humans: Role of oxidative stress. Journal of Physiology, 590(21), 5519–5528. 10.1113/jphysiol.2012.236992 22907057 PMC3515835

[phy214585-bib-0022] Jouett, N. P. , Moralez, G. , White, D. W. , Eubank, W. L. , Chen, S. , Tian, J. , Smith, M. L. , Zimmerman, M. C. , & Raven, P. B. (2016). N‐Acetylcysteine reduces hyperacute intermittent hypoxia‐induced sympathoexcitation in human subjects. Experimental Physiology, 101(3), 387–396. 10.1113/EP085546 27027616 PMC4817372

[phy214585-bib-0023] Kety, S. S. , & Schmidt, F. C. (1946). Effects of alterations in the arterial tensions of carbon dioxide and oxygen on cerebral blood flow and cerebral oxygen consumption of normal young men. Journal of Clinical Investigation, 5(1), 484–492. 10.1172/JCI101995 PMC439519 16695569

[phy214585-bib-0024] Lavi, S. , Gaitini, D. , Milloul, V. , & Jacob, G. (2006). Impaired cerebral C02 vasoreactivity: Association with endothelial dysfunction. American Journal of Physiology. Heart and Circulatory Physiology, 291, H1856–1861. 10.1152/ajpheart.00014.2006 16766649

[phy214585-bib-0044] Lin, B.‐H. (2014). USDA ERS ‐ ERSs food consumption and nutrient intake data—tools for assessing Americans diets (2014). Retrieved from https://www.ers.usda.gov/amber‐waves/2014/october/erss‐food‐consumption‐and‐nutrient‐intake‐data‐tools‐for‐assessing‐americans‐diets/ (Accessed: 3 March 2020).

[phy214585-bib-0025] Lombard, J. H. , Sylvester, F. A. , Phillips, S. A. , & Frisbee, J. C. (2003). High‐salt diet impairs vascular relaxation mechanisms in rat middle cerebral arteries. American Journal of Physiology. Heart and Circulatory Physiology, 284(4), H1124–H1133. 12456391 10.1152/ajpheart.00835.2002

[phy214585-bib-0026] Markus, H. , & Cullinane, M. (2001). Severely impaired cerebrovascular reactivity predicts stroke and TIA risk in patients with carotid artery stenosis and occlusion. Brain, 124(3), 457–467. 10.1093/brain/124.3.457 11222446

[phy214585-bib-0027] Marley, C. J. , Hodson, D. , Brugniaux, J. V. , Fall, L. , & Bailey, D. M. (2017). Post‐prandial hyperlipidaemia results in systemic nitrosative stress and impaired cerebrovascular function in the aged. Clinical Science, 131, 2807–2812. 10.1042/CS20171406 29054860

[phy214585-bib-0028] Matic, A. , Jukic, I. , Stupin, A. , Baric, L. , Mihaljevic, Z. , Unfirer, S. , Tartaro Bujak, I. , Mihaljevic, B. , Lombard, J. H. , & Drenjancevic, I. (2018). High salt intake shifts the mechanisms of flow‐induced dilation in the middle cerebral arteries of Sprague‐Dawley rats. American Journal of Physiology. Heart and Circulatory Physiology, 315, H718–H730. 10.1152/ajpheart.00097.2018 29906224

[phy214585-bib-0029] McDonnell, M. N. , Berry, N. M. , Cutting, M. A. , Keage, H. A. , Buckley, J. D. , & Howe, P. R. C. (2013). Transcranial Doppler ultrasound to assess cerebrovascular reactivity: Reliability, reproducibility and effect of posture. PeerJ, 1, e65 10.7717/peerj.65 23646284 PMC3642776

[phy214585-bib-0030] Migdal, K. U. , Robinson, A. T. , Watso, J. C. , Babcock, M. C. , Serrador, J. M. , & Farquhar, W. B. (2020). A high‐salt meal does not augment blood pressure responses during maximal exercise. Applied Physiology, Nutrition and Metabolism, 45(2), 123–128. 10.1139/apnm-2019-0217 31238011

[phy214585-bib-0031] NDSR (2012). Nutrition Coordinating Center. .

[phy214585-bib-0032] Patik, J. C. , Tucker, W. J. , Curtis, B. M. , Nelson, M. D. , Nasirian, A. , Park, S. , & Brothers, R. M. (2018). Fast‐food meal reduces peripheral artery endothelial function but not cerebral vascular hypercapnic reactivity in healthy young men. Physiological Reports, 6(18), e13867. 30221831 10.14814/phy2.13867 PMC6139709

[phy214585-bib-0033] Petrica, L. , Petrica, M. , Vlad, A. , Bob, F. , Gluhovschi, C. , Gluhovschi, G. , Jianu, C. D. , Ursoniu, S. , Schiller, A. , Velciov, S. , Trandafirescu, V. , & Bozdog, G. (2007). Cerebrovascular reactivity is impaired in patients with non‐insulin‐dependent diabetes mellitus and microangiopathy. Wiener Klinische Wochenschrift, 119(11–12), 365–371. 10.1007/s00508-007-0809-0 17634895

[phy214585-bib-0034] Purkayastha, S. , & Sorond, F. (2012). Transcranial Doppler ultrasound: Technique and application. Seminars in Neurology, 32(4), 411–420. 10.1055/s-0032-1331812 23361485 PMC3902805

[phy214585-bib-0035] Ramick, M. G. , Brian, M. S. , Matthews, E. L. , Patik, J. C. , Seals, D. R. , Lennon, S. L. , Farquhar, W. B. , & Edwards, D. G. (2019). Apocynin and Tempol ameliorate dietary sodium‐induced declines in cutaneous microvascular function in salt‐resistant humans. American Journal of Physiology. Heart and Circulatory Physiology, 317(1), H97–H103. 10.1152/ajpheart.00786.2018 31074652 PMC6692730

[phy214585-bib-0036] Ringelstein, E. B. , Sievers, C. , Ecker, S. , Schneider, P. A. , & Otis, S. M. (1988). Noninvasive assessment of co2‐induced cerebral vasomotor response in normal individuals and patients with internal carotid artery occlusions. Stroke, 19(8), 963–979. 10.1161/01.str.19.8.963 3135641

[phy214585-bib-0037] Serrador, J. M. , & Freeman, R. (2017). Enhanced cholinergic activity improves cerebral blood flow during orthostatic stress. Frontiers in Neurology, 8, 1–9. 10.3389/fneur.2017.00103 28373858 PMC5357636

[phy214585-bib-0038] Serrador, J. M. , Picot, P. A. , Rutt, B. K. , Shoemaker, J. K. , & Bondar, R. L. (2000). MRI measures of middle cerebral artery diameter in conscious humans during stimulated orthostasis. Stroke, 31, 1672–1678. 10.1161/01.STR.31.7.1672 10884472

[phy214585-bib-0039] Serrador, J. M. , Schlegel, T. T. , Black, F. O. , & Wood, S. J. (2005). Cerebral hypoperfusion precedes nausea during centrifugation. Aviation Space and Environmental Medicine, 76(2), 91–96. PMC2792728 15742822

[phy214585-bib-0040] Settakis, G. , Páll, D. , Molnár, C. , Bereczki, D. , Csiba, L. , & Fülesdi, B. (2003). Cerebrovascular reactivity in hypertensive and healthy adolescents: TCD with vasodilatory challenge. Journal of Neuroimaging, 13(2), 106–112. 10.1111/j.1552-6569.2003.tb00166.x 12722492

[phy214585-bib-0041] Silvestrini, M. et al (2000). Impaired cerebral vasoreactivity and risk of stroke in patients with asymptomatic carotid artery stenosis. JAMA, 283(16), 2122–2127. 10.1001/jama.283.16.2122 10791504

[phy214585-bib-0042] Suckling, R. J. , He, F. J. , Markandu, N. D. , & MacGregor, G. A. (2012). Dietary salt influences postprandial plasma sodium concentration and systolic blood pressure. Kidney International, 81(4), 407–411. 10.1038/ki.2011.369 22048126

[phy214585-bib-0043] Thijssen, D. H. J. , Bruno, R. M. , van Mil, A. C. C. M. , Holder, S. M. , Faita, F. , Greyling, A. , Zock, P. L. , Taddei, S. , Deanfield, J. E. , Luscher, T. , Green, D. J. , & Ghiadoni, L. (2019). Expert consensus and evidence‐based recommendations for the assessment of flow‐mediated dilation in humans. European Heart Journal, 40, 2534–2547. 10.1093/eurheartj/ehz350 31211361

[phy214585-bib-0014] U.S. Department of Health and Human Services and U.S. Department of Agriculture . (2015). 2015–2020 Dietary Guidelines for Americans (8th ed.). Retrieved from https://health.gov/dietaryguidelines/2015/guidelines/ (Accessed: 20 June 2018).

[phy214585-bib-0045] Wasserman, A. J. , & Patterson, J. L. (1961). The cerebral vascular response to reduction in arterial carbon dioxide tension. Journal of Clinical Investigation, 40(7), 1297–1303. 10.1172/JCI104359 13783303 PMC290842

[phy214585-bib-0046] Whelton, P. K. , Appel, L. J. , Sacco, R. L. , Anderson, C. A. M. , Antman, E. M. , Campbell, N. , Dunbar, S. B. , Frohlich, E. D. , Hall, J. E. , Jessup, M. , Labarthe, D. R. , MacGregor, G. A. , Sacks, F. M. , Stamler, J. , Vafiadis, D. K. , & Van Horn, L. V. (2012). Sodium, blood pressure, and cardiovascular disease. Circulation, 126(24), 2880–2889. 10.1161/CIR.0b013e318279acbf 23124030

[phy214585-bib-0047] Whelton, P. K. , Carey, R. M. , Aronow, W. S. , Casey, D. E. , Collins, K. J. , Dennison Himmelfarb, C. , DePalma, S. M. , Gidding, S. , Jamerson, K. A. , Jones, D. W. , MacLaughlin, E. J. , Muntner, P. , Ovbiagele, B. , Smith, S. C. , Spencer, C. C. , Stafford, R. S. , Taler, S. J. , Thomas, R. J. , Williams, K. A. , … Wright, J. T. (2018). 2017 ACC/AHA/AAPA/ABC/ACPM/AGS/APhA/ASH/ASPC/NMA/PCNA guideline for the prevention, detection, evaluation, and management of high blood pressure in adults. Journal of the American College of Cardiology, 71(19), e127–e248. 10.1016/j.jacc.2017.11.006 29146535

